# Application of a New Hybrid Model with Seasonal Auto-Regressive Integrated Moving Average (ARIMA) and Nonlinear Auto-Regressive Neural Network (NARNN) in Forecasting Incidence Cases of HFMD in Shenzhen, China

**DOI:** 10.1371/journal.pone.0098241

**Published:** 2014-06-03

**Authors:** Lijing Yu, Lingling Zhou, Li Tan, Hongbo Jiang, Ying Wang, Sheng Wei, Shaofa Nie

**Affiliations:** School of Public Health, Tongji Medical College, Huazhong University of Science and Technology, Wuhan, China; University of Illinois at Chicago, United States of America

## Abstract

**Background:**

Outbreaks of hand-foot-mouth disease (HFMD) have been reported for many times in Asia during the last decades. This emerging disease has drawn worldwide attention and vigilance. Nowadays, the prevention and control of HFMD has become an imperative issue in China. Early detection and response will be helpful before it happening, using modern information technology during the epidemic.

**Method:**

In this paper, a hybrid model combining seasonal auto-regressive integrated moving average (ARIMA) model and nonlinear auto-regressive neural network (NARNN) is proposed to predict the expected incidence cases from December 2012 to May 2013, using the retrospective observations obtained from China Information System for Disease Control and Prevention from January 2008 to November 2012.

**Results:**

The best-fitted hybrid model was combined with seasonal ARIMA 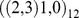 and NARNN with 15 hidden units and 5 delays. The hybrid model makes the good forecasting performance and estimates the expected incidence cases from December 2012 to May 2013, which are respectively −965.03, −1879.58, 4138.26, 1858.17, 4061.86 and 6163.16 with an obviously increasing trend.

**Conclusion:**

The model proposed in this paper can predict the incidence trend of HFMD effectively, which could be helpful to policy makers. The usefulness of expected cases of HFMD perform not only in detecting outbreaks or providing probability statements, but also in providing decision makers with a probable trend of the variability of future observations that contains both historical and recent information.

## Introduction

Hand-foot-mouth disease (HFMD) is a common acute infectious disease, which is featured by fever, painful sores in the mouth, and a rash with blisters on the hands, feet and buttocks [1], [2]. The dominant strain is Coxsackievirus A16 (CA 16) and Enterovirus A71 (EV 71). HFMD occurs mainly among children under 5 years of age, who are at the greatest risk. The infection is typically mild and self-limiting with few complications [3]. However, severe cases with complications of central nervous system, always caused by EV 71 [4], would occasionally lead to neurological sequelae or subsequent quick death.

Since the 1970s, epidemics of HFMD have been reported worldwide in Hungary, southeastern Australia, United States, England, Wales, Malaysia and Singapore [5]–[10]. In China, after its first emergence in Shanghai in 1981 [11], several sporadic cases were reported in Beijing, Tianjin, Jilin, Guangdong and other provinces [12]–[18]. In 2008, an unprecedented large-scale epidemic of HFMD broke out first in Fuyang of Anhui province [19]. The persistent outbreaks sounded the alarm bell to the Chinese government. Therefore, on May 2, 2008, the Ministry of Health of the People's Republic of China added HFMD to its category ‘C’ of notifiable diseases, which means all the cases must be reported through a national web-based system named China Information System for Diseases Control and Prevention (CISDCP) built in 2004 [20].

Statistics show that in 2008, among the category ‘C’ of notifiable diseases [21], the number of reported cases of HFMD ranked second after other infectious diarrhea but the reported deaths ranked first. However, both the reported cases and deaths of HFMD had always ranked the top among the category ‘C’ of notifiable diseases from 2009 to 2012 [22]–[25]. To tackle the growing threat, the government and the public health officials were aware of the importance of early detection of outbreaks, early recognition, early intervention in the disease and commencing aggressive therapy to minimize the impact exerted by HFMD.

Recently, researchers are interested in forecasting the incidence of infectious disease, using the liner time series forecasting models such as seasonal auto-regressive integrated moving average (ARIMA) models [26]–[30]. However, most real time series always contain nonlinear structures, from which liner models cannot yield adequate results [31]. To fit the nonlinear structures, nonlinear models such as artificial neural networks (ANNs), bilinear, auto-regressive conditional heteroskedasticity models (ARCH) performed better than liner models. Among them, ANNs have flexible nonlinear function mapping capability, which can approximatecontinuous measurable function with arbitrarily expected accuracy [32], so that nonlinear structures can performed well [33]. However, when it comes to real time series that contain liner and nonlinear structures, neither linear nor nonlinear models seems to be satisfactory. To solve this problem, this paper attempts to use hybrid models combining liner and nonlinear models to improve the prediction accuracy by taking the advantages of both models. These hybrid models have been found to be viable contenders to various traditional time series models [31], [34]–[37].

Considering the variety of influencing factors on HFMD, this paper proposes a new hybrid model combining seasonal ARIMA and nonlinear auto-regressive neural network (NARNN) to predict the incidence of HFMD in Shenzhen. The aim of this paper is to describe the future trend of this disease and to achieve the early detection and early warning by mathematical method.

## Method

### Setting

Shenzhen is the first – and one of the most successful – Special Economic Zones (SEZs) in China as well as the largest manufacturing base in the world. Because of this, Shenzhen becomes the largest migrant city in China with a population of roughly ten million in 2010 Census. About six million are migrant workers who return their homes on weekends or festivals, and live in factory dormitories during the workdays. Therefore, it's difficult to obtain the exact annually average statistical population. Instead, this paper used the numbers of incidence cases as an evaluation indicator.

### Data resource

This paper used the incidence cases of HFMD during January 2008 to August 2012 as the training data, the data during September 2012 to November 2012 as the validation set, and the data during December 2012 to May 2013 as the forecasting set. All the data were obtained from CISDCP mentioned above. The CISDCP has two important improvements compared with the previous reporting system [20]. The first is that diseases are reported in real time, which allows public-health officials to have real-time information and also helps to reduce the under-reporting of infectious diseases. The second improvement includes the availability of case-based reporting instead of aggregate reporting, which immediately helps public-health officials comprehensively identify the characteristics, nature and location of a particular disease outbreak or clusters of cases. In addition, a series of measures have been taken to improve the quality of data reporting, such as annual field audits, national training conferences on data entry, routine quality checking and quality reporting of data at varied levels of medical institutions. A crucial reason for the research to consider is that the data is viewed as being of high quality with respect to accuracy, comparability, timeliness, relevance and usability under the category ‘C’ of notifiable diseases management for HFMD.

### Statistical method

#### The Auto-regressive Integrated Moving Average Models

Given a stationary time series of data 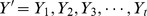, an auto-regressive moving average (ARMA) model is respectively composed by two parts, an auto regressive (AR) part of order p and a moving average (MA) part of order q.

An AR model of order p, denoted by AR 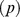, is given by
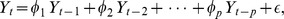



an MA model of order q, denoted by MA 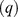, is given by
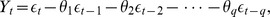



an ARMA model of order p and q, denoted by ARMA 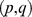, is given by
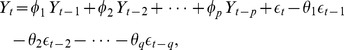
where 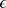 is the random error term assumed to be independent and referred as a white noise identically distributed with a mean of zero and equal variance [38]. It's usually interpreted as external effect that the model can't explain. Stationarity occurs in a time series when the mean value of the series remains constant over the time series. Frequently, differencing is needed to achieve stationarity in the model. It is denoted by ARIMA 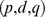, where d is the value of differencing orders. In addition, a top priority of the model building is to identify the appropriate model order 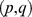 [31]. Box and Jenkins [38] proposed to identify the order of the model by the autocorrelation function (ACF) and the partial autocorrelation function (PACF) as the basic tool. If monthly data were used in the analysis, periodicity of series would be shown, which was more likely to lead to useful forecasting and should cover at least 2 periods [29]. Seasonal terms are also incorporated into ARIMA model, which are denoted by seasonal ARIMA 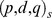, where S is often referred as the value of per period. The residuals, the differences between each observation and prediction according to the model, should also be inspected, ideally small and show no secular or seasonal trend [29].

#### The Artificial Neural Networks

ANNs consist of a large number of highly connected nonlinear simple unit and store information in the connections between units by self-learning and self-organizing [39]. The commonest type of ANNs is the single hidden layerback-propagation (BP) neural network, which is a kind of multilayered feedforward neural network. The studied process of the BP neural network is formed by two parts: signal forward-propagating and error signal reverse dissemination, and the input single spreading from the input layer, captured by the hidden layer passing on to the output layer. When expected output value can't be obtained from the output layer, the process turns to error signal reverse dissemination stage, and with the back-propagation of the error is repeated, the error signal reduces and the correct response rate rises [40]. In this paper, NARNN, which is a dynamic neural network based on the BP neural network with the feedback layers to approximate the non-linear function [39], is applied. The main differences in the design process occur because the inputs to the dynamic network are time sequences so it is good at time series forecasting.

#### The hybrid models with seasonal ARIMA and NARNN

In this section, the hybrid model building procedures will be described step by step in detail. In the seasonal ARIMA model stage, the main aim is to extract the linear information. In the beginning, one or more abnormal observations (AO) based on the real events, which could explain the reasons of abnormalities, would be found. Each abnormal observation is replaced with a missing value, and filled by the function of SAS ‘expand’ procedure. Next in the identified step, ACF and PACF could be detected visually by examining a regression line scatterplot, so that the AR and MA components and s would be identified with possible values. If the data is non-stationary, regular differencing or seasonal differencing is needed then, and the value of d and s would be the orders of differencing. Augmented Dickey-Fuller Unit Root (ADF) test is used to identify whether the series after differencing is stationary or not. Once the orders are specified, estimation of the model parameters by least squares estimation is thus straightforward. The parameters with significant statistical difference are reserved and the others are excluded. The next step of model building is the diagnostic checking of model adequacy. This is basically to check whether the model assumptions are satisfied or not. If the model were not adequate, a new tentative model would be identified. The steps of parameter estimation and model verification are not stopped until the new tentative model is satisfied. The autocorrelation and partial autocorrelation of residuals help to verify whether the series of residuals to be the white noise by using Boxing-Ljung Q-test. In the last step, the one with the lowest Bayesian Information Criterion (BIC) value is chosen to be the best-fitted model. P<0.05 is considered statistically significant. The training set is used to build the model and get 3 steps forecasting for validation, and the performance is evaluated by validation set. After that, the observations of the training set and validation set are utilized to build a new model again, repeating the same modeling procedures. All ARIMA modeling is implemented via SAS9.2 system.

In NARNN stage, the main aim is to model the nonlinear and probable linear relationships existing in the residuals of linear modeling and the original series. To model the NARNN, it is generally best to start with the neural network time series tool (ntstool) in the MATLAB, which can automatically generate command-line scripts in accordance with the demand of the research. First, the target series is inputted to obtain the command-line script and the next 6 months data set for multi-step-ahead prediction. Then the division of data using the provided divider and function, which divides the data into contiguous blocks, respectively 80% of the target series for training, 10% for validation and 10% for testing, is set up. In the last step, the number of hidden neurons and feedback delays are adjusted by trial and error, based on the error autocorrelation plot, the time series response plot and the Mean Square Error (MSE) of training and testing data, to select the optimal model. With this tool, the other important parameters are set as the defaults, such as the tan-sigmoid transfer function in the hidden layer, the linear transfer function in the output layer and the Levenberg-Marquardtal training-algorithm.

Based on the adjusted residuals, the expected monthly incidence cases of HFMD can be obtained. The eventual predictions are the sum of seasonal ARIMA predictions and adjusted residuals. It is 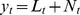, where 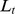 denotes the predictions of linear model and 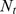 denotes the residuals adjusted by nonlinear model.

## Results

### Cases distribution and demographic characteristics

During the study period, the reported cases of HFMD in Shenzhen increased every year with a slight rise in 2009 and a dramatic rise since 2010 (Table 1). The amounts of severe cases and fatal cases in each year were parallel with the reported cases except a significant decrease in 2012. The male predominance was found in each year, and the proportion of male to female remained stable. The age distribution was similar every year, and children <5 years old were under greatest risk, especially those between 1 to 3 years old. The annual number of the cases occurring among different child-care centers showed little change. The majority of patients were home-cared, with kindergarten-cared and school-cared as the second and third respectively. The results of laboratory diagnosis showed that the first dominant strain of the epidemic in Shenzhen was EV 71, and there was a significant increasing trend of other virus supplanting Cox A16 to be the second dominant strain.

**Table 1:** Case distribution and demographic characteristics of HFMD in Shenzhen from January 2008 to November 2012.

	2008	2009	2010	2011	2012*
**Number of cases**	7149	9121	23288	24838	30021
**Number of severe cases**	4	37	92	150	55
**Number of fatal cases**	1	5	6	6	2
**Gender**					
Male	4574(63.98%)	5802(63.61%)	14564(62.54%)	15818(63.68%)	18763
Female	2575(36.02%)	3319(36.39%)	8724(37.46%)	9020(36.32%)	11258
**Age**					
0∼	635(8.88%)	1037(11.37%)	2518(10.81%)	3143(12.65%)	3943(13.13%)
1∼	1083(15.15%)	1529(16.76%)	6657(28.59%)	7692(30.97%)	9219(30.71%)
2∼	1718(24.03%)	2462(26.99%)	4988(21.42%)	5087(20.48%)	5793(19.30%)
3∼	1398(19.56%)	1892(20.74%)	4208(18.07%)	4386(17.66%)	5130(17.09%)
4∼	1132(15.83%)	1060(11.62%)	2220(9.53%)	2241(9.02%)	3022(10.07%)
>5	1183(16.55%)	1141(12.51%)	2697(11.58%)	2289(9.22%)	2914(9.70%)
**Form of child care**					
Home care	4633(64.81%)	6886(75.50%)	17147(73.63%)	19002(76.50%)	22343(74.42%)
Kindergarten care	2078(29.07%)	1827(20.03%)	5033(21.61%)	4850(19.53%)	6553(21.83%)
School care	364(5.09%)	279(3.06%)	878(3.77%)	779(3.14%)	888(2.96%)
**Type of pathogen**					
Number of laboratory diagnosis	157	86	169	142	55
EV 71	107(68.15%)	64(74.42%)	111(65.68%)	107(75.35%)	38(69.09%)
CA 16	50(31.85%)	21(24.42%)	57(33.73%)	16(11.27%)	5(9.09%)
Others	0(0.00%)	1(1.16%)	1(0.59%)	19(13.38%)	12(21.82%)

### Trend and seasonality of HFMD epidemic

**Figure 1:**
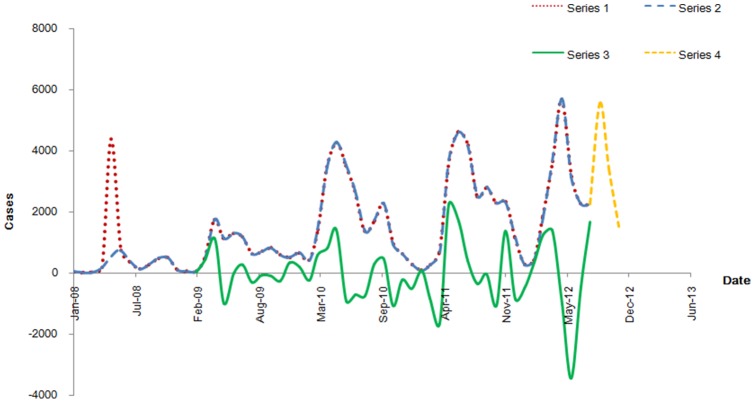
Series of observations of HFMD in Shenzhen. Series 1 shows the observations of the training set (from January 2008 to August 2012). Series 2 shows the observations of training set without the abnormal observation (AO). Series 3 shows the series 2 achieving stationary after one regular differencing and one seasonal differencing (d = 1, s = 12). Series 4 shows the validation set (from September 2012 to November 2012).

The monthly numbers of reported cases of HFMD were graphically shown in Figure 1 with an increasing trend, a clear yearly periodicity and significant fluctuations in its yearly mean. During the study period, the highest peaks of seasonal periodicity occurred in April and remained high until July; the second small peaks appeared during September to November. From November to February of the following year, the incidence of HFMD was at a low level until the next epidemic started.

### Results of the validation set

**Figure 2:**
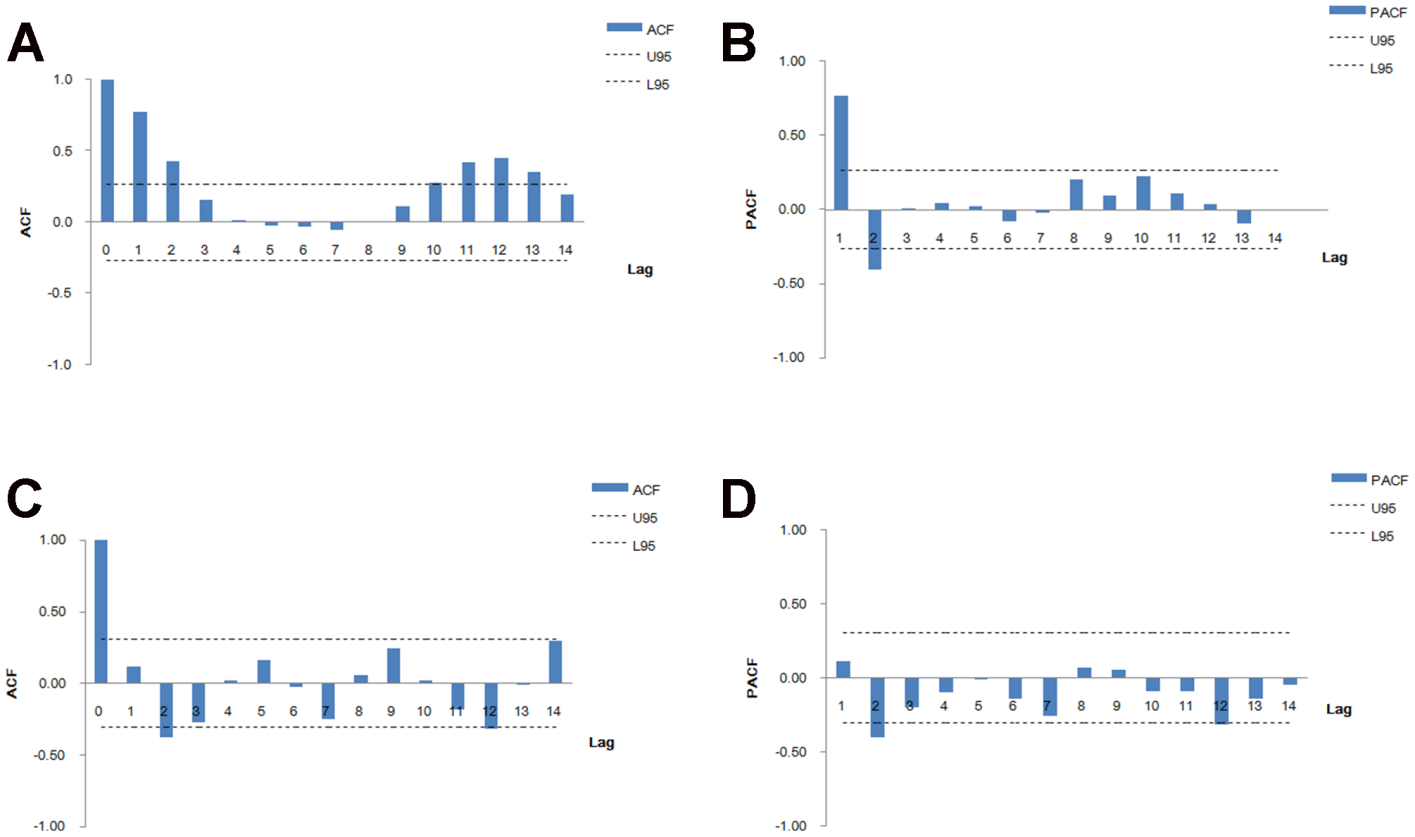
Autocorrelation function (ACF) and partial autocorrelation function (PACF) plotted against time lags. A and B show ACF and PACF of the training set. C and D show ACF and PACF of the training set after one order of regular differencing and one order of seasonal differencing (d = 1, s = 12). After differencing, Most of the correlations fall around zero within their 95% confidence intervals (95%CI, U95: upper limit of 95%CI, L95: lower limit of 95%CI) except the one at the first lag, which is indicated the series would achieve stationary.

In our opinion, due to the upgrade of HFMD to the category ‘C’ of notifiable diseases by the Ministry of Health of the PRC in May 2008, and the inclusion in CISDCP, the value in May 2008 is considered as an AO, which could be explained by increasing efforts in detecting and reporting HFMD. The differencing series appears stationary with a same mean value and variance over time (Figure 1). This suggests that it would be appropriate to consider an order d = 1 and S = 12 in the fitted model given by seasonal ARIMA 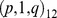. We could get the same conclusion by graphing the ACF and PACF (Figure 2). After the steps of parameter estimation and model verification, the model with order (2, 0) is best fitted to the data (Table 2). The final mathematical form of the seasonal ARIMA model is ARIMA 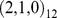.

**Table 2:** Parameter estimation and model verification of seasonal ARIMA model with minimum BIC Value (3, 0)  = 13.732.

Lag	Parameter	Estimate	*P-value*	Q(18)**	*P-value*	Estimate'	*P-value*	Q(18)'**	*P-value*
0	MU	−6.780	0.941	13.04	0.599	-	-	-	-
1	AR 1,1	0.093	0.552			-	-	-	-
2	AR 1,2	−0.387	0.013*			−0.406	0.008*	18.16	0.379
3	AR 1,3	−0.313	0.096			-	-	-	-

* Parameter estimation was considered statistically significant (P<0.05).

**Box-Ljung test at lag 18 for the series of residuals.

**Figure 3:**
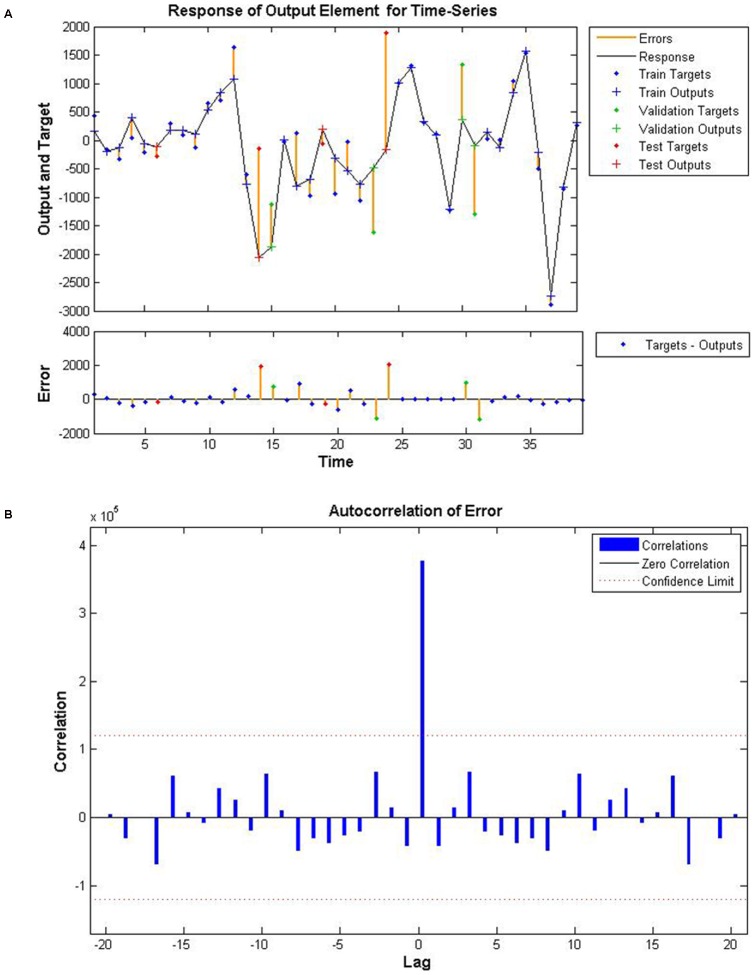
Time series response and error autocorrelation plot in training, testing and validation set of NARNN. A shows the time series response in training, testing and validation set. B shows the error autocorrelation plot in training, testing and validation set. In B, all the correlations fall within the 95% confidence limits around zero except the one at zero lag, which is indicated the model would be adequate.

The optimum NARNN this paper proposed to apply to forecast the residuals series produced by best-fitted seasonal ARIMA model has 12 hidden units and 4 delays. The MSE of training, validation, and testing data subsets are 8.2037×10^4^, 1.0477×10^6^, and 1.9939×10^6^ respectively. The correlations of prediction errors fall within the 95% confidence limits around zero, therefore the model is adequate for the data (Figure 3).

**Figure 4:**
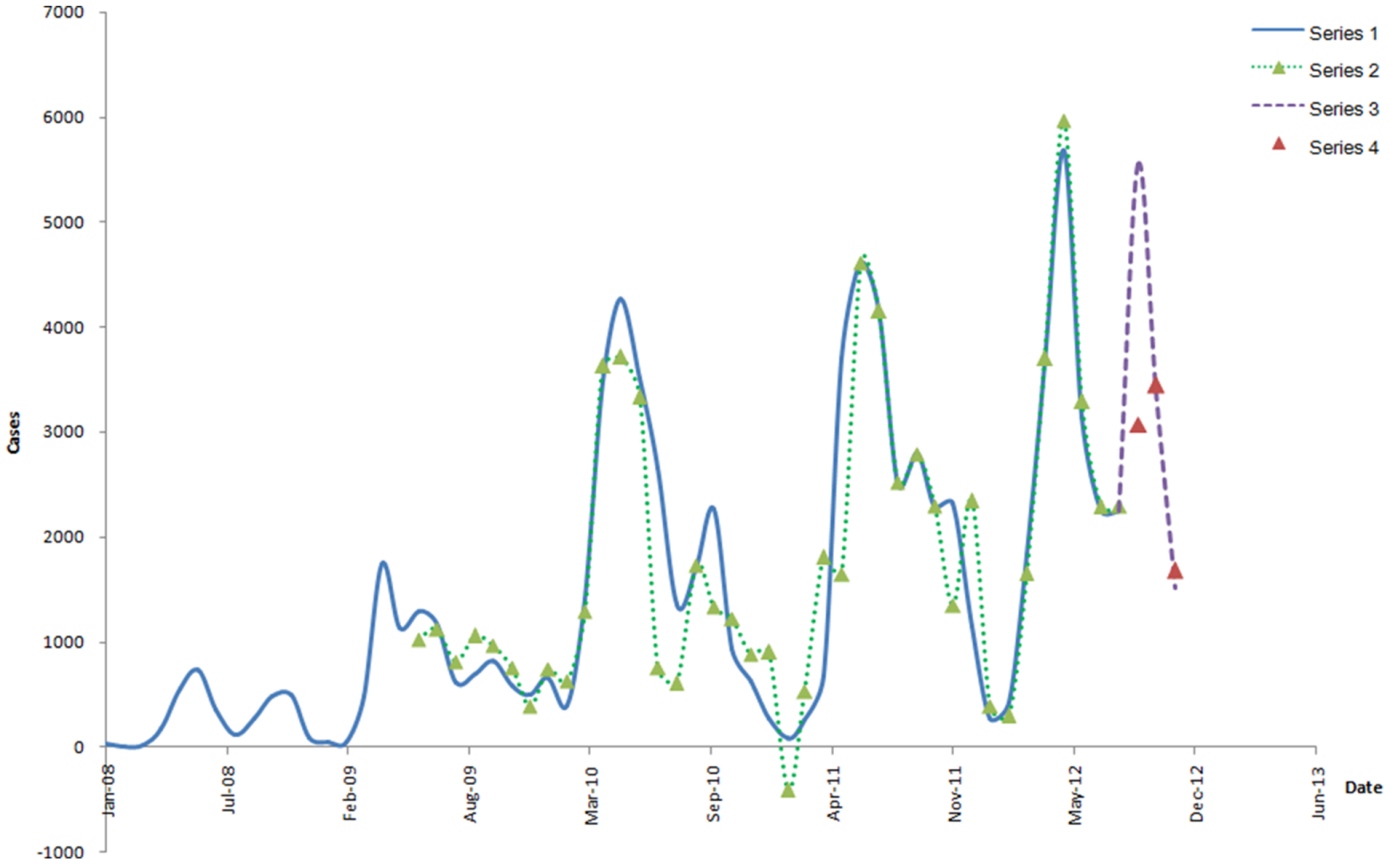
Series of the predictions of the validation set. Series 1 shows the observations of training set without the abnormal observation. Series 3 shows the validation set. Series 2 shows the predictions of training set obtained by hybrid model combined with ARIMA 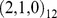 and NARNN with 12 hidden units and 4 delays, and Series 4 shows the predictions of validation set obtained by the same model. Each prediction is very close to each observation.

After model building, the predictions of the validation set are thus obtained (Figure 4), which are very close to the observations and indicate that the proposed model was fitted and made good performance.

### Results of forecasting set

**Figure 5:**
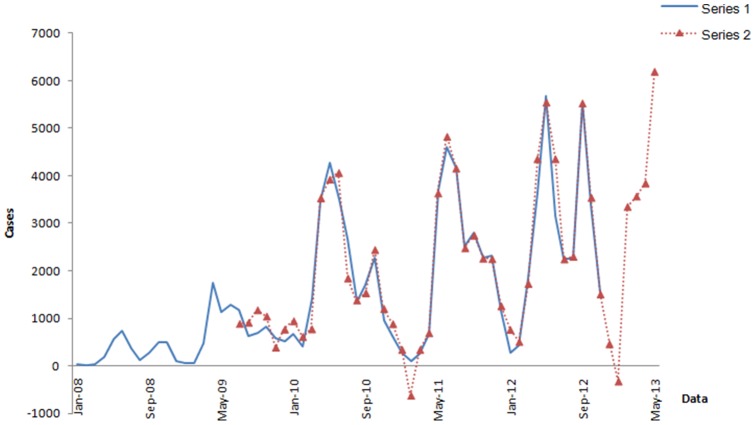
Series of predictions of all the observations. Series 1 shows the observations of the training set without the abnormal observations and the validation set. Series 2 shows the predictions of series 1 and the expected cases of forecasting set (from December 2012 to May 2013) obtained by ARIMA 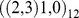 and NARNN with 15 hidden units and 5 delays. There is a significantly increasing trend in first half of 2013.

The best-fitted hybrid model of all the observations is with a combination of ARIMA 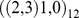 and NARNN with 15 hidden units and 5 delays, and the predictions of forecasting set are shown in Figure 5. It is easy to find out that each prediction obtained from the hybrid model is very close to each observation. The value of February 2011 is a negative value (−181.166) (Table 3), and the value of the observation at the same time is 84, which indicates that if observations were extremely small, the corresponding predictions would be negative. Therefore, the trough of the epidemic in 2013 would occur in January. In addition, compared with previous years, the expected incidence cases in 2013 would rise rapidly, and the first peak would begin in February (Table 2). The second peak would occur in April and the highest in May. The amount of total expected cases would be higher than any other previous years.

**Table 3:** Expected incidence cases and observations in the corresponding period from 2010 to 2013 in Shenzhen.

Month	Observations			Expected cases
	2010	2011	2012	2013
December*	504	631	1154	−965.03
January	658	276	271	−1879.58
February	402	84	426	4138.26
March	1415	252	1843	1858.17
April	3481	675	3659	4061.86
May	4267	3702	5684	6163.16
Total	10727	5620	13037	16221.45**

*December in the previous year.

**We made the assumption that the expected cases in January and February were zero.

## Discussion

During the five-year study period, HFMD cases in Shenzhen increased every year. However, during the H1N1 pandemic in 2009, preventive measures such as massive use of face masks, school closures and reduction of outdoor activities could partly explain why there were the lower HFMD incidence cases, which was similar with the situation in Guangzhou [41]. The numbers of severe cases and fatal cases dropped significantly in 2012, which indicated that the measures on controlling severe and fatal cases were playing their roles. The found of male predominance was similar with the results of Zeng's study in Shanghai [42] and Zhu's study in mainland China [43], which could be attributed to more restlessness and more opportunities for boys to contract the disease compared with girls. Patients with HFMD were mostly aged 1∼3 years old, which was similar with previous study [3], [42]–[45]. The majority of patients were home-care which was different from situations in Shanghai [42]. A possible reason is that the proportion of migrant workers in Shenzhen substantially outnumbered that in Shanghai and migrant parents don't have enough time to take care of their children. Moreover, they are generally less educated and under poorer living conditions or financial status, which makes them the high-risk group of infection. The most commonly isolated enterovirus of HFMD cases were types associated with EV 71, which was similar with situations in mainland China [46]. However, the reported cases of infection by other enterovirus increased significantly, and public health officials should alert the outbreaks of new types of HFMD. The seasonal distribution in Shenzhen was similar to the southern region of China [46].

The new hybrid model is applied to forecast the incidence cases of HFMD, and the results show that the combination model could be an effective way for prediction. In previous studies, multivariate SARIMA models with some meteorological variables were built to achieve a better predicting performance of statistical methods [41]. Nevertheless, the epidemic of HFMD is influenced not only by meteorological factors [41], [47]–[50], but also by total population and population density, rural versus urban living, literacy, enterovirus positivity, sanitary conditions and population susceptibility, even other unknown factors [46]. The hybrid model, whose aim is to reduce the risk of using an inappropriate model and which obtain more accurate results, takes advantages of the unique strength of seasonal ARIMA and NARNN in linear and nonlinear modeling. In such a model, the seasonal ARIMA model fits the non-stationary linear component, whilst the neural network model fits nonlinearity [31].

When the epidemics or outbreaks occur, infectious disease should be investigated and the causes of them fully understood. However, this is impossible under any circumstances for scientific and practical reasons. Referring to experience, the usefulness of forecasting expected cases of HFMD performs not only in detecting outbreaks or providing probability statements, but also in giving decision makers a probable trend of the variability of future observations that contains both historical and recent information. As Table 3 shows, the comparison implies that the pandemic would increase earlier than the previous years, and the incidence population would come to a bigger size. The expected incidence trend is proposed to provide them with predicting future trend, early forecasting and detecting peak time and scale of HFMD outbreaks when observations significantly exceed standard thresholds, and evaluating effectiveness of health measures when the observations are lower than the forecasting trend, so that they can improve surveillance, make prevention and control strategies, and allocate health resources. In the practice, the key point is to keep the forecasts at hand completely, ideally on display, and to write in new observations to update the data as soon as they become available [29], especially when new control measures are taken, or else, the model would have no chance to help detect epidemic or outbreaks sooner than otherwise possible. At the same time, the method is simple and easy to get started, which can be applied availably to field epidemiological investigation, for it only requires investigators to have a computer.

However, some flawed parts may affect the outcomes. The foundation of model building is the data reported to CISDCP, and the quality of reported cases everyday directly influence the forecasting performance of the model. Good quality of data collection by this system has been demonstrated by a recent data quality inspection report, apart from the following problems [51]. The reported data of HFMD, collected retrospectively when doctors investigate patients, would be inaccurate. The reported data would not be comprehensive because some mildly affected patients may not go to any medical institution for treatment, and those patients are not reported to the system. Lastly, a minor increase in the number of reported cases would occur due to the enhancive consciousness of the importance of reporting HFMD in medical institutions by HFMD-related policies. In addition, the hybrid models are combined with the liner model and the nonlinear model, thus they have the disadvantages of both linear and nonlinear models. Since the ANN models belong to the blackbox type of models, it may limit the model's ability to extrapolate beyond its training data as well as the implementation of subjective initiatives by operators in ANN analysis.
